# Coronavirus induces diabetic macrophage-mediated inflammation via SETDB2

**DOI:** 10.1073/pnas.2101071118

**Published:** 2021-09-03

**Authors:** William J. Melvin, Christopher O. Audu, Frank M. Davis, Sriganesh B. Sharma, Amrita Joshi, Aaron DenDekker, Sonya Wolf, Emily Barrett, Kevin Mangum, Xiaofeng Zhou, Monica Bame, Alex Ruan, Andrea Obi, Steven L. Kunkel, Bethany B. Moore, Katherine A. Gallagher

**Affiliations:** ^a^Section of Vascular Surgery, Department of Surgery, University of Michigan, Ann Arbor, MI 48109;; ^b^Department of Internal Medicine, University of Michigan, Ann Arbor, MI 48109;; ^c^Department of Pathology, University of Michigan, Ann Arbor, MI 48109;; ^d^Department of Microbiology and Immunology, University of Michigan, Ann Arbor, MI 48109

**Keywords:** coronavirus, diabetes, inflammation, epigenetics, monocyte/macrophage

## Abstract

COVID-19 induces a robust, extended inflammatory “cytokine storm” that contributes to an increased morbidity and mortality, particularly in patients with type 2 diabetes (T2D). Macrophages are a key innate immune cell population responsible for the cytokine storm that has been shown, in T2D, to promote excess inflammation in response to infection. Using peripheral monocytes and sera from human patients with severe acute respiratory syndrome coronavirus 2 (SARS-CoV-2), and a murine hepatitis coronavirus (MHV-A59) (an established murine model of SARS), we identified that coronavirus induces an increased Mφ-mediated inflammatory response due to a coronavirus-induced decrease in the histone methyltransferase, SETDB2. This decrease in SETDB2 upon coronavirus infection results in a decrease of the repressive trimethylation of histone 3 lysine 9 (H3K9me3) at NFkB binding sites on inflammatory gene promoters, effectively increasing inflammation. Mφs isolated from mice with a myeloid-specific deletion of SETDB2 displayed increased pathologic inflammation following coronavirus infection. Further, IFNβ directly regulates SETDB2 in Mφs via JaK1/STAT3 signaling, as blockade of this pathway altered SETDB2 and the inflammatory response to coronavirus infection. Importantly, we also found that loss of SETDB2 mediates an increased inflammatory response in diabetic Mϕs in response to coronavirus infection. Treatment of coronavirus-infected diabetic Mφs with IFNβ reversed the inflammatory cytokine production via up-regulation of SETDB2/H3K9me3 on inflammatory gene promoters. Together, these results describe a potential mechanism for the increased Mφ-mediated cytokine storm in patients with T2D in response to COVID-19 and suggest that therapeutic targeting of the IFNβ/SETDB2 axis in T2D patients may decrease pathologic inflammation associated with COVID-19.

## Significance

The COVID-19 pandemic has disproportionately affected patients with comorbidities, namely, obesity and type 2 diabetes. Macrophages (Mφs) are a key innate immune cell primarily responsible for the harmful, hyperinflammatory “cytokine storm” in patients that develop severe COVID-19. We describe a mechanism for this Mφ-mediated cytokine storm in response to coronavirus. In response to coronavirus infection, expression of the chromatin-modifying enzyme, SETDB2, decreases in Mφs, leading to increased transcription of inflammatory cytokines. Further, we find SETDB2 is regulated by an interferon beta (IFNβ)/JaK/STAT3 mechanism, and that exogenous administration of IFNβ can reverse inflammation, particularly in diabetic Mφs via an increase in SETDB2. Together, these results suggest therapeutic targeting of the IFNβ/SETDB2 axis in diabetic patients with COVID-19 may decrease pathologic inflammation.

COVID-19, caused by infection with the severe acute respiratory syndrome coronavirus 2 (SARS-CoV-2), has caused an estimated 3.93 million deaths worldwide as of June 2021 (1). One of the hallmarks of severe COVID-19 is a hyperimmune response that results in an inflammatory “cytokine storm” (2). Poor outcomes are generally ascribed to the development of this cytokine storm, and elevated serum levels of TNFα, IL-6, and IL-8 are independent predictors of COVID-19 disease severity and death (3–5). The inflammatory cytokine storm in COVID-19 is thought to be primarily mediated by macrophages (Mφs), as the cytokine profile in patients with severe disease mirrors other cytokine release syndromes driven by Mφs (6–8). In viral infections that result in less severe disease, a kinetic balance between proinflammatory and regulatory Mφ phenotypes must be achieved in order to trigger the adaptive immune response necessary for effective clearance of the virus (9). Highly pathogenic respiratory viruses such as SARS-CoV-1, Middle East respiratory syndrome (MERS)-CoV, influenza, and respiratory syncytial virus are adept at inciting a prolonged inflammatory Mφ phenotype (9), resulting in a massive cytokine and chemokine release, allowing for direct viral infection of infiltrating cells. Continued virally mediated Mφ dysfunction leads to massive cell death of these inflammatory Mφs, and damage of alveolar lung tissue (9), increasing morbidity for the patient. The factors that trigger Mφs to develop a hyperinflammatory cytokine storm in COVID-19 are not completely understood but may involve a defective interferon response (10) or specific patient comorbidities that change Mφ responsiveness to the SARS-CoV-2 virus (6).

COVID-19 severity has been directly linked to obesity and type 2 diabetes (T2D) (11–15). Causes of this increased susceptibility of patients with T2D to severe SARS-CoV-2 infection are likely multifactorial (16); however, patients with T2D often develop an inflammatory cytokine storm (3), due to unclear etiology.

Epigenetic regulation of gene expression plays a major role in the function of immune cells in both normal and pathologic conditions and in response to viral insult by controlling downstream protein expression patterns (17, 18). It is well established that histone modifications regulate immune profile and cytokine expression (18–21) in Mφs in response to injury or infection, yet the specific mechanisms underlying how these modifications contribute to the cytokine storm in patients with severe COVID-19 is unknown. One such histone modification, methylation of lysine 9 on histone 3 (H3K9), condenses chromatin and prevents transcription factor access, effectively silencing transcription. We and others have found that the enzyme SETDB2, which trimethylates H3K9, is critical for regulating Mφ-mediated inflammation in wound repair (22). However, there remains a paucity of data regarding epigenetic mechanisms that regulate Mφ phenotypes and inflammation after viral infection.

Here, we examined inflammation in human and murine Mφs in the context of infection with SARS-CoV-2 and a murine hepatitis coronavirus, MHV-A59. Following coronavirus infection, the histone methyltransferase SETDB2 was decreased in normal and diabetic Mφs. Loss of SETDB2 with coronavirus infection led to increased production of inflammatory cytokines (IL-1β, TNFα, and IL-6) in Mφs via alterations in H3K9me3 at NFkB binding sites on inflammatory gene promoters following infection. Experiments in myeloid-specific murine models deficient in SETDB2 revealed that SETDB2 facilitated the inflammatory response in Mφs in response to coronavirus infection. Further, SETDB2 expression in Mφs was regulated by IFNβ via the JaK1/STAT3 pathway. Levels of IFNβ were decreased in plasma from COVID-19 (+) human patients with T2D compared to non-T2D COVID-19 (+) patients, as well as in diabetic murine Mφs. Administration of IFNβ to Mφs infected with coronavirus increased *Setdb2* expression to a greater degree in the setting of diabetes, and decreased transcription of inflammatory genes. Our findings have therapeutic implications for abrogating the cytokine storm associated with COVID-19, particularly in T2D patients.

## Results

### The Murine Coronavirus MHV-A59 Induces Mφ-Mediated Inflammation.

**Fig. 1.**
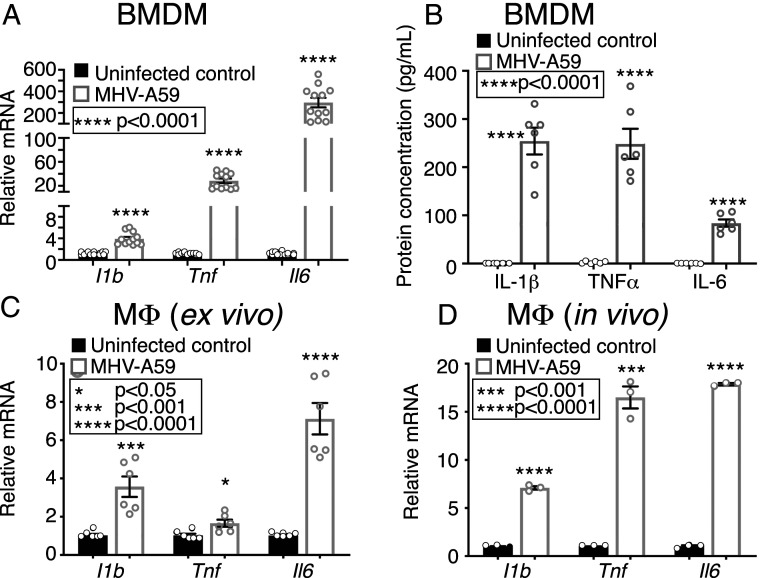
The murine coronavirus MHV-A59 induces Mφ-mediated inflammation. (*A*) *Il1b, Tnf,* and *Il6* expression measured in BMDMs from *C57BL/6* mice 24 h following in vitro infection with MHV-A59 (MOI 0.5) and compared to uninfected BMDMs (*n* = 12 mice per group, run in triplicate). (*B*) Supernatant protein levels of IL-1β, TNFα, and IL-6 from BMDMs from *C57BL/6* mice 24 h following in vitro infection with MHV-A59 (MOI 0.5) and compared to uninfected BMDMs (*n* = 6 mice per group, run in triplicate). (*C*) *Il1b, Tnf,* and *Il6* expression measured in splenic Mφs (CD3^−^/CD19^−^/NK1.1^−^/Ly6G^−^/CD11b^+^) from C57BL/6 mice 24 h following ex vivo infection with MHV-A59 (MOI 0.5) and compared to uninfected Mφs (*n* = 5 mice per group, pooled and run in triplicate). (*D*) *Il1b, Tnf,* and *Il6* expression measured in splenic Mφs (CD3^−^/CD19^−^/NK1.1^−^/Ly6G^−^/CD11b^+^) isolated from *C57BL/6* mice 3 d after intranasal infection with MHV-A59 (2 × 10^5^ pfu) compared to uninfected Mφs (*n* = 5 mice per group, run in triplicate). *P < 0.05, ***P < 0.001, ****P < 0.0001. Data are presented as the mean ± SEM. All data are representative of two to four independent experiments. Data were first analyzed for normal distribution, and, if data passed the normality test, two-tailed Student’s *t* test was used.

It is well established that severe cases of COVID-19 induce a robust, extended inflammatory response associated with a profound cytokine storm that contributes to increased morbidity and mortality (3). Mφs are a key innate immune cell responsible for this cytokine storm (6–8). In order to mechanistically define how coronavirus affects Mφs leading to this pathologic inflammation, we examined infection of murine Mφs with murine hepatitis virus A59 (MHV-A59). Prior studies have found that expression of key inflammatory cytokines known to be increased during infection with SARS-CoV-1 (IL-1β) and SARS-CoV-2 (TNFα and IL-6) (6) is increased during infection with MHV-A59 as well (23). First, bone marrow Mφs (BMDMs) from normal *C57BL/6* mice were infected in vitro (multiplicity of infection [MOI] 0.5) for 4 h, and cytokine expression at postinfection was measured using qPCR (Fig. 1*A*). Protein levels in the supernatant were also measured using enzyme-linked immunosorbent assay (ELISA) (Fig. 1*B*). Compared to uninfected controls, MHV-A59 induced IL-1β, TNFα, and IL-6 expression and cytokine production. When higher MOI was used (i.e., MOI 1.0), an increase in cellular apoptosis and necrosis was seen compared to uninfected BMDMs, as measured by Annexin/propidium iodide staining (*SI Appendix*, Fig. S1). Next, in order to examine the effect of MHV-A59 on isolated Mφs treated ex vivo, splenic Mφs (CD3^−^/CD19^−^/NK1.1^−^/Ly6G^−^/CD11b^+^) were infected (MOI 0.5) for 4 h, and subsequent cytokine expression was analyzed. Compared to uninfected controls, MHV-A59 induced *Il1b*, *Tnf*, and *Il6* expression (Fig. 1*C*). To determine whether these effects occur in vivo, *C57BL/6* mice were infected with respiratory MHV-A59 (2 × 10^5^ plaque-forming units [pfu]). Five and seven days after infection, Mφs (CD3^−^/CD19^−^/NK1.1^−^/Ly6G^−^/CD11b^+^) were harvested and compared to uninfected controls. These in vivo Mφs also demonstrated increased expression of *Il1b*, *Tnf*, and *Il6* at both time points postinfection (Fig. 1*D* and *SI Appendix*, Fig. S2). Furthermore, lung histology taken from the same infected mice showed acute inflammation and perivascular leukocytic infiltration (*SI Appendix*, Fig. S3), suggesting that MHV-A59 can serve as a model to mechanistically study the in vivo effects of coronavirus on inflammation in Mφs. Taken together, these results provide evidence that MHV-A59 can induce inflammatory cytokine expression in Mφs both in vitro and in vivo.

### The Histone Methyltransferase SETDB2 Is Decreased in Human and Murine Mφs following Infection with SARS-CoV-2 and MHV-A59.

**Fig. 2.**
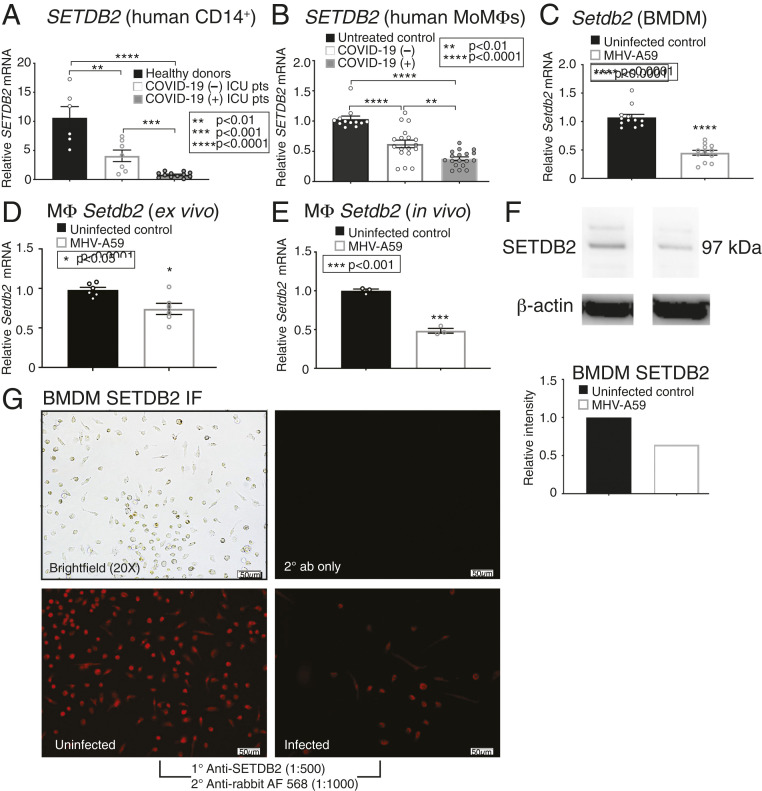
The histone methyltransferase SETDB2 is decreased in human and murine Mφs following infection with SARS-CoV-2 or MHV-A59. (*A*) *SETDB2* expression measured in human CD14^+^ cells sorted from peripheral blood from healthy donors (*n* = 6) and COVID-19 (+) (*n* = 12) and COVID-19 (−) (*n* = 7) critically ill patients, run in triplicate. (*B*) *SETDB2* expression measured in MoMs from healthy donors (*n* = 4) 24 h following exposure to serum (1:1 diluted in RPMI) from COVID-19 (+) (*n* = 18) and COVID-19 (−) (*n* = 18) critically ill patients, or untreated (*n* = 12), run in triplicate). (*C*) *Setdb2* expression measured in BMDMs from *C57BL/6* mice 5 h following in vitro infection with MHV-A59 (MOI 0.5) and compared to uninfected BMDMs (*n* = 12 mice per group, run in triplicate). (*D*) *Setdb2* expression measured in splenic Mφs (CD3^−^/CD19^−^/NK1.1^−^/Ly6G^−^/CD11b^+^) from *C57BL/6* mice 5 h following ex vivo infection with MHV-A59 (MOI 0.5) and compared to uninfected Mφs (*n* = 5 mice per group, pooled and run in triplicate, repeated twice). (*E*) Setdb2 expression measured in splenic Mφs (CD3^−^/CD19^−^/NK1.1^−^/Ly6G^−^/CD11b^+^) isolated from *C57BL/6* mice 5 d after intranasal infection with MHV-A59 (2 × 10^5^ pfu) compared to uninfected Mφs (*n* = 5 mice per group, pooled and run in triplicate). (*F*) SETDB2 protein measured in BMDMs from *C57BL/6* mice 12 h following in vitro infection with MHV-A59 (MOI 0.5) via Western blot (*n* = 5 mice per group, pooled). Representative blots are shown. (*G*) SETDB2 protein immunofluorescent microscopy in BMDMs from *C57BL/6* mice 12 h following in vitro infection with MHV-A59 (MOI 0.5) (*n* = 5 mice/group, pooled). Representative images are shown. *P < 0.05, **P < 0.01, ***P < 0.001, ****P < 0.0001. Data are presented as the mean ± SEM. All data are representative of two to four independent experiments. Data were first analyzed for normal distribution, and, if data passed the normality test, two-tailed Student’s *t* test was used.

Our group has previously identified that epigenetic alterations can underlie Mφ-mediated inflammation in human disease (24–28). Given that we recently identified that SETDB2, a histone methyltransferase that trimethylates H3K9 (H3K9me3) and represses gene expression and NFkB-dependent inflammatory gene promoters, can control Mφ-mediated inflammation in the setting of wound repair (22), we investigated whether SETDB2 or other epigenetic enzymes known to influence Mφ inflammation (i.e., JMJD3, MOF, and MLL1 [KMT2A]) (24–26, 28–30) were altered in Mφs in response to coronavirus infection. Peripheral blood was collected from critically ill, ICU patients with and without COVID-19, and CD14^+^ monocytes were sorted and analyzed for SETDB2. We found that CD14^+^ monocytes from patients positive for COVID-19 demonstrated significantly reduced expression of *SETDB2* compared to other critically ill ICU patients without COVID-19 and to healthy donors (Fig. 2*A*). SETDB2 protein was also decreased in CD14^+^ monocytes from COVID-19 (+) patients (*SI Appendix*, Fig. S4). Further, sera were collected from critically ill, ICU patients infected with SARS-CoV-2. These sera had no detectable SARS-CoV-2 RNA (*SI Appendix*, Fig. S5*A*), but demonstrated a significantly decreased IFNβ compared to sera taken from critically ill ICU uninfected (control) patients (*SI Appendix*, Fig. S5*B*). These sera were then added 1:1 with fresh media onto monocyte-derived Mφs (MoMs) from healthy donors (22). We found that MoMs treated with sera from patients infected with SARS-CoV-2 demonstrated markedly reduced expression of *SETDB2* (Fig. 2*B*) compared to MoMs treated with sera from critically ill COVID-19 (−) patients. Our previous work demonstrated that SETDB2 is up-regulated by type I interferons (22). Given that there is increasing evidence of an inappropriate, inadequate type I interferon response in COVID-19 (6, 10), and that we found that sera from COVID-19 (+) patients had decreased IFNβ compared to sera from uninfected ICU patients, we postulate that this lack of IFNβ led to decreased expression of *SETDB2* in Mφs, with subsequent loss of repressive H3K9me3 at NFkB-dependent inflammatory cytokine promoters, and thus an unrestricted Mφ-mediated inflammatory cytokine storm associated with coronavirus infection. In vitro BMDMs, as well as ex vivo infected Mφs and in vivo isolated Mφs, from wild-type (WT) *C57BL/6* mice all demonstrated decreased *Setdb2* expression in response to MHV-A59 infection compared to uninfected controls (Fig. 2 *C*–*E*), and further analysis of infected BMDMs demonstrated decreased SETDB2 protein with infection (Fig. 2 *F* and *G*). We also examined expression of other key epigenetic enzymes in BMDMs and found no significant changes associated with coronavirus infection (*SI Appendix*, Fig. S6). This decrease in *SETDB2* is in contrast to influenza infection, where previous work (18, 31) has shown an increase in *SETDB2.* This decrease leaves patients susceptible to bacterial superinfection, a phenomenon not overly common in COVID-19 or other coronavirus infections (32, 33).

### SETDB2 Regulates Inflammatory Cytokines IL-1β, TNFα, and IL-6 via H3K9me3 at NFκB Promoters in Response to Infection with Coronavirus MHV-A59.

**Fig. 3.**
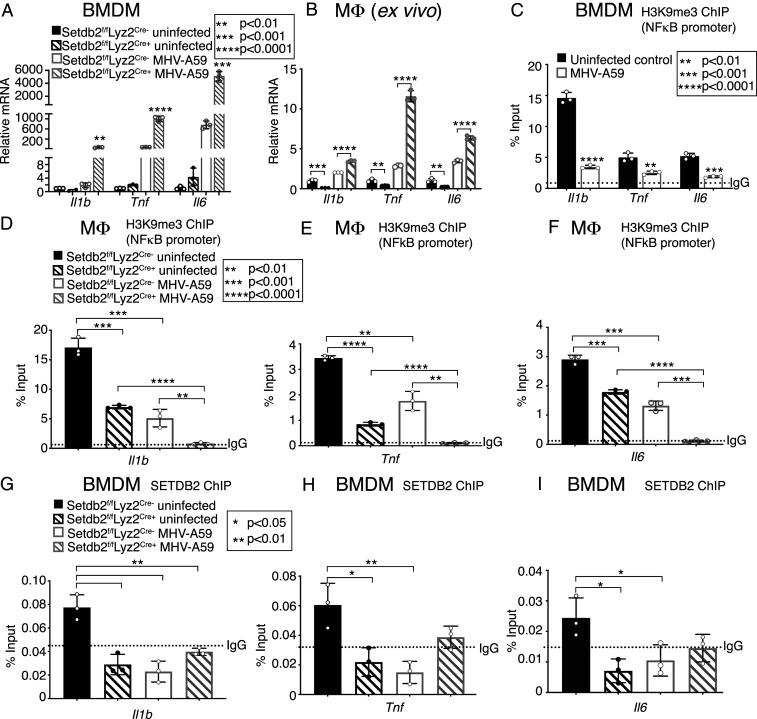
SETDB2 regulates inflammatory cytokines IL-1β, TNFα, and IL-6 via H3K9me3 at NFκB binding sites on gene promoters in response to infection with coronavirus MHV-A59. (*A*) *Il1b, Tnf,* and *IL6* expression measured in BMDMs from *Setdb2*^*f/f*^*Lyz2*^*Cre+*^ mice 24 h following in vitro infection with MHV-A59 (MOI 0.5) and compared to infected *Setdb2*^*f/f*^*Lyz2*^*Cre-*^ BMDMs (*n* = 5 mice per group, pooled and run in triplicate). (*B*) *Il1b, Tnf,* and *IL6* expression measured in splenic Mφs (CD3^−^/CD19^−^/NK1.1^−^/Ly6G^−^/CD11b^+^) from *Setdb2*^*f/f*^*Lyz2*^*Cre+*^ mice 12 h following ex vivo infection with MHV-A59 (MOI 0.5) and compared to *Setdb2*^*f/f*^*Lyz2*^*Cre-*^ Mφs (*n* = 5 mice per group, pooled and run in triplicate). (*C*) ChIP analysis of H3K9me3 on the *Il1b, Tnf,* and *IL6* promoters in BMDMs from *C57BL/6* mice 24 h following in vitro infection with MHV-A59 (MOI 0.5) and compared to uninfected BMDMs (*n* = 5 mice per group, pooled and run in triplicate). (*D*–*F*) ChIP analysis of H3K9me3 on the *Il1b* (*D*), *Tnf* (*E*), and *Il6* (*F*) promoters splenic Mφs (CD3^−^/CD19^−^/NK1.1^−^/Ly6G^−^/CD11b^+^) from *Setdb2*^*f/f*^*Lyz2*^*Cre+*^ mice and *Setdb2*^*f/f*^*Lyz2*^*Cre-*^ littermate controls 12 h following ex vivo infection with MHV-A59 (MOI 0.5) (*n* = 5 mice per group, pooled and run in triplicate). (*G*–*I*) ChIP analysis of SETDB2 on the *Il1b* (*G*), *Tnf* (*H*), and *Il6* (*I*) promoters in BMDMs from *Setdb2*^*f/f*^*Lyz2*^*Cre+*^ mice and *Setdb2*^*f/f*^*Lyz2*^*Cre-*^ littermate controls mice 5 h following in vitro infection with MHV-A59 (MOI 0.5) (*n* = 5 mice per group, pooled and run in triplicate). *P < 0.05, **P < 0.01, ***P < 0.001, ****P < 0.0001. Data are presented as the mean ± SD. All data are representative of two to four independent experiments. Data were first analyzed for normal distribution, and, if data passed the normality test, two-tailed Student’s *t* test was used.

Given our findings of decreased *SETDB2* expression in human and murine Mφs in response to coronavirus infection, we examined the role of Mφ-specific *Setdb2* deficiency on Mφ-mediated inflammation in response to coronavirus MHV-A59. First, BMDMs isolated from mice deficient in SETDB2 in myeloid cells (*Setdb2*^*f/f*^*Lyz2*^*Cre+*^) and littermate controls (*Setdb2*^*f/f*^*Lyz2*^*Cre-*^) were infected with MHV-A59 (MOI 0.5). Mφs deficient in *Setdb2* showed significantly increased expression of inflammatory cytokines (*Il1b*, *Tnf*, and *IL6*) at 24 h postinfection compared to *Setdb2*^*f/f*^*Lyz2*^*Cre-*^ controls (Fig. 3*A*). This relationship was also seen ex vivo, when splenic Mφs (CD3^−^/CD19^−^/NK1.1^−^/Ly6G^−^/CD11b^+^) isolated from *Setdb2*^*f/f*^*Lyz2*^*Cre+*^ mice and littermate controls were infected with MHV-A59 (MOI 0.5) (Fig. 3*B*). Next, a chromatin precipitation (ChIP) assay for H3K9me3 on the NFkB binding sites of proinflammatory gene promoters was performed on BMDMs infected with MHV-A59. Decreased H3K9me3 was found on the NFkB binding promoters of inflammatory genes following infection, corresponding to the decreased *Setdb2* expression seen after MHV-A59 infection (Fig. 3*C*). As SETDB2 is not the only epigenetic enzyme capable of altering the methylation status at H3K9, a ChIP assay was performed in splenic Mφs (CD3^−^/CD19^−^/NK1.1^−^/Ly6G^−^/CD11b^+^) isolated from *Setdb2*^*f/f*^*Lyz2*^*Cre+*^ mice and littermate controls infected with MHV-A59. Mφs deficient in *Setdb2* failed to trimethylate H3K9 at inflammatory gene promoters, especially following coronavirus infection (Fig. 3 *D*–*F*). We further examined the role of SETDB2 in H3K9me3 by performing a SETDB2-ChIP analysis of BMDMs from *Setdb2*^*f/f*^*Lyz2*^*Cre+*^ mice and littermate controls infected with MHV-A59. This demonstrated decreased SETDB2 at inflammatory gene promoters both following MHV-A59 infection and in SETDB2 myeloid cell−deficient BMDMs both before and after MHV-A59 infection (Fig. 3 *G*–*I*). Taken together, these results suggest that SETDB2-mediated H3K9me3 regulates inflammatory gene expression in Mφs during coronavirus infection.

### IFNβ/JaK1/STAT-3 Signaling Regulates *Setdb2* Expression in Mφs in Response to Infection with Coronavirus MHV-A59.

**Fig. 4.**
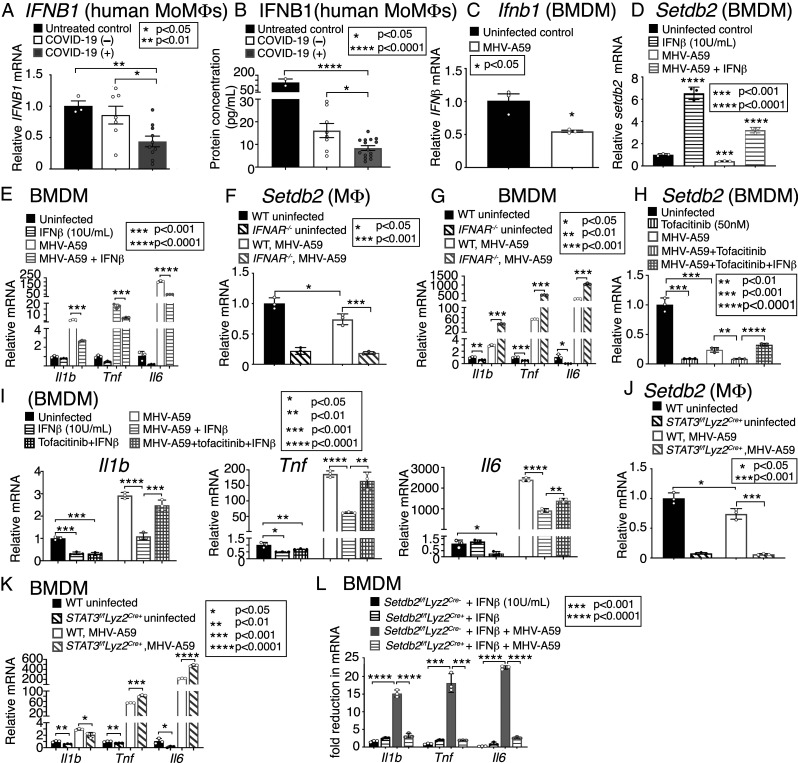
IFNβ/JaK1/STAT-3 signaling regulates Setdb2 expression in Mφs in response to infection with coronavirus MHV-A59. (*A*) *IFNB1* expression measured in MoMs from healthy donors (*n* = 3) 24 h following exposure to serum (1:1 diluted in RPMI) from COVID-19 (+) (*n* = 10) and COVID-19 (−) (*n* = 8) critically ill patients, or untreated (*n* = 3, run in triplicate). (*B*) Supernatant IFNβ protein measured in MoMs from healthy donors (*n* = 3) 24 h following exposure to serum (1:1 diluted in RPMI) from COVID-19 (+) (*n* = 15) and COVID-19 (−) (*n* = 8) critically ill patients, or untreated (activated with 100 ng/mL LPS) (*n* = 2, run in triplicate). (*C*) *Ifnb1* expression measured in BMDMs from *C57BL/6* mice 5 h following in vitro infection with MHV-A59 (MOI 0.5) (*n* = 5 mice per group, pooled and run in triplicate). (*D*) *Setdb2* expression measured in BMDMs from *C57BL/6* mice 5 h following in vitro infection with MHV-A59 (MOI 0.5), with and without coadministration with IFNβ (10 U/mL), compared to uninfected BMDMs (*n* = 5 mice per group, pooled and run in triplicate). (*E*) *Il1b, Tnf,* and *IL6* expression measured in BMDMs from *C57BL/6* mice 24 h following in vitro infection with MHV-A59 (MOI 0.5), with and without coadministration with IFNβ (10 U/mL), compared to uninfected BMDMs (*n* = 5 mice per group, pooled and run in triplicate). (*F*) *Setdb2* expression measured in splenic Mφs (CD3^−^/CD19^−^/NK1.1^−^/Ly6G^−^/CD11b^+^) from *Ifnar*^*−/−*^ mice 5 h following ex vivo infection with MHV-A59 (MOI 0.5), compared to Mφs from *Ifnar*^*+/+*^ littermate controls (*n* = 5 mice per group, pooled and run in triplicate). (*G*) *Il1b, Tnf,* and *IL6* expression measured in BMDMs from *Ifnar*^*−/−*^ mice 24 h following in vitro infection with MHV-A59 (MOI 0.5), compared to BMDMs from *Ifnar*^*+/+*^ littermate controls (*n* = 5 mice per group, pooled and run in triplicate). (*H*) *Setdb2* expression measured in BMDMs from *C57BL/6* mice 5 h following in vitro infection with MHV-A59 (MOI 0.5), with and without coadministration with tofacitinib (50 nM) and IFNβ (10 U/mL) with tofacitinib (*n* = 5 mice per group, pooled and run in triplicate). (*I*) *Il1b, Tnf,* and *IL6* expression in BMDMs from *C57BL/6* mice 24 h following in vitro infection with MHV-A59 (MOI 0.5), with and without coadministration with tofacitinib (50 nM) and IFNβ (10 U/mL) with tofacitinib (*n* = 5 mice per group, pooled and run in triplicate). (*J*) *Setdb2* expression measured in splenic Mφs (CD3^−^/CD19^−^/NK1.1^−^/Ly6G^−^/CD11b^+^) from *Stat3*^*f/f*^*Lyz2*^*Cre+*^ mice 5 h following ex vivo infection with MHV-A59 (MOI 0.5), compared to infected Mφs from *Stat3*^*f/f*^*Lyz2*^*Cre-*^ littermate controls (*n* = 5 mice per group, pooled and run in triplicate). (*K*) *Il1b, Tnf,* and *IL6* expression measured in BMDMs from *Stat3*^*f/f*^*Lyz2*^*Cre+*^ mice 24 h following in vitro infection with MHV-A59 (MOI 0.5), compared to BMDMs from *Stat3*^*f/f*^*Lyz2*^*Cre-*^ littermate controls (*n* = 5 mice per group, pooled and run in triplicate). (*L*) *Il1b, Tnf,* and *IL6* expression measured in BMDMs from *Setdb2*^*f/f*^*Lyz2*^*Cre+*^ mice 24 h following in vitro infection with MHV-A59 (MOI 0.5), coadministered IFNβ (10 U/mL) and compared to infected *Setdb2*^*f/f*^*Lyz2*^*Cre-*^ BMDMs coadministered IFNβ (*n* = 5 mice per group, pooled and run in triplicate). *P < 0.05, **P < 0.01, ***P < 0.001, ****P < 0.0001. Data are presented as the mean ± SEM (in *A* and *B*) and mean ± SD (in *C*–*L*). All data are representative of two to four independent experiments. Data were first analyzed for normal distribution, and, if data passed the normality test, two-tailed Student’s *t* test was used.

Given that there is evidence of an inadequate type I interferon response in COVID-19 (6, 10), that the sera from COVID-19 (+) patients had less IFNβ than COVID-19 (−) ICU patients, and that our previous work has shown that IFNβ up-regulates *Setdb2* during wound repair (22), we hypothesized that this pathway may be relevant in coronavirus infection. Human MoMs treated with sera from critically ill COVID-19 (+) patients were found to have less IFNβ messenger RNA (mRNA) and protein expression than those treated with sera from critically ill COVID-19 (−) patients or untreated MoMs (Fig. 4 *A* and *B*). Infected BMDMs from *C57BL/6* mice also showed decreased *Ifnb1* expression upon MHV-A59 infection (Fig. 4*C*). BMDMs were then isolated and treated for 4 h as follows: 1) uninfected/untreated control, 2) 10 U/mL IFNβ, 3) 0.5 MOI MHV-A59, or 4) IFNβ + MHV-A59. We found that IFNβ (10 U/mL) can up-regulate *Setdb2* expression during coronavirus infection, reversing the effects of MHV-A59 that serve to decrease *Setdb2* (Fig. 4*D*). This up-regulation of *Setdb2* in infected BMDMs treated with IFNβ correlated with a significant reduction in inflammatory cytokine (*Il1b*, *Tnf*, and *Il6*) expression (Fig. 4*E*). To investigate whether expression of *Setdb2* during coronavirus is dependent on IFNβ signaling, we next utilized *Ifnar*^*−/−*^ mice, which lack the receptor for IFNαβ. When *Ifnar*^*−/−*^ Mφs (CD3^−^/CD19^−^/NK1.1^−^/Ly6G^−^/CD11b^+^) were isolated and infected with MHV-A59 (MOI 0.5), *Setdb2* expression was significantly less than in infected control, IFNαβ receptor intact (*Ifnar*^*+/+*^) Mφs (Fig. 4*F*). BMDMs from these *Ifnar*^*−/−*^ mice also exhibited increased expression of *Il1b*, *Tnf*, and *Il6* with MHV-A59 infection compared to uninfected *Ifnar*^*+/+*^ controls (Fig. 4*G*). Since signaling of IFNβ via the receptor for IFNαβ is well known to activate JAK1/STAT-3 signal transduction cascades (34, 35), we then examined whether JaK1 or STAT3 inhibition would alter *Setdb2* expression and enhance proinflammatory gene expression in Mφs during coronavirus infection. BMDMs from *C57BL/6* mice were isolated and infected with MHV-A59 and treated with either the JaK1,3 inhibitor, tofacitinib (50 nM) alone or with IFNβ and tofacitinib together. Treatment with tofacitinib demonstrated a decrease in *Setdb2* expression, and IFNβ in the presence of tofacitinib could not up-regulate *Setdb2*, suggesting IFNβ regulates *Setdb2* via Jak1 (Fig. 4*H*). Tofacitinib also reduced the antiinflammatory effect of IFNβ on these BMDMs (Fig. 4*I*). Similarly, when BMDMs were isolated from mice deficient in STAT3 in myeloid cells (*Stat3*^*f/f*^*Lyz2*^*Cre+*^ mice), and infected with MHV-A59, *Setdb2* expression was significantly decreased and inflammatory cytokines were increased compared to littermate controls (Fig. 4 *J* and *K*). The decreased expression of *Setdb2* with JaK1/STAT3 inhibition demonstrates the role of JaK1/STAT3 signaling in regulating *Setdb2* expression during coronavirus infection. We found that IFNβ administration to MHV-A59−infected BMDMs decreases inflammatory cytokine expression; however, this is not entirely surprising, given that this has previously been shown in SARS-CoV-1 and MERS, although the mechanisms concerning these IFNβ-induced changes in inflammatory gene expression were unknown (36). In order to specifically examine whether IFNβ regulation of Setdb2 alters inflammatory cytokine expression given the pleiotropic nature of IFNβ and JAK/STAT signaling, BMDMs from *Setdb2*^*f/f*^*Lyz2*^*Cre+*^ mice and littermate controls (*Setdb2*^*f/f*^*Lyz2*^*Cre-*^) were infected with MHV-A59 and treated with IFNβ (10 U/mL). BMDMs from littermate controls demonstrated a 5- to 10-fold reduction in inflammatory cytokine expression in this experiment compared to Setdb2-deficient BMDMs (Fig. 4 *J*). Similarly, these *Setdb2*^*f/f*^*Lyz2*^*Cre+*^ display increased levels (∼12-fold) of *Ifnb1* compared to littermate controls both before and especially after coronavirus infection, suggesting a possible feedback attempt to increase *Setdb2* expression in these deficient BMDMs (*SI Appendix*, Fig. S7). Taken together, these results indicate that the IFNβ-mediated reduction in Mφ inflammation associated with coronavirus infection is due to alterations in *Setdb2* via an IFNβ-induced JaK1/STAT-3 pathway.

### Reduced Expression of SETDB2 Mediates an Increased Inflammatory Cytokine Response in Human and Murine Diabetic Mφs in Response to Infection with Coronavirus SARS-CoV-2 and MHV-A59.

**Fig. 5.**
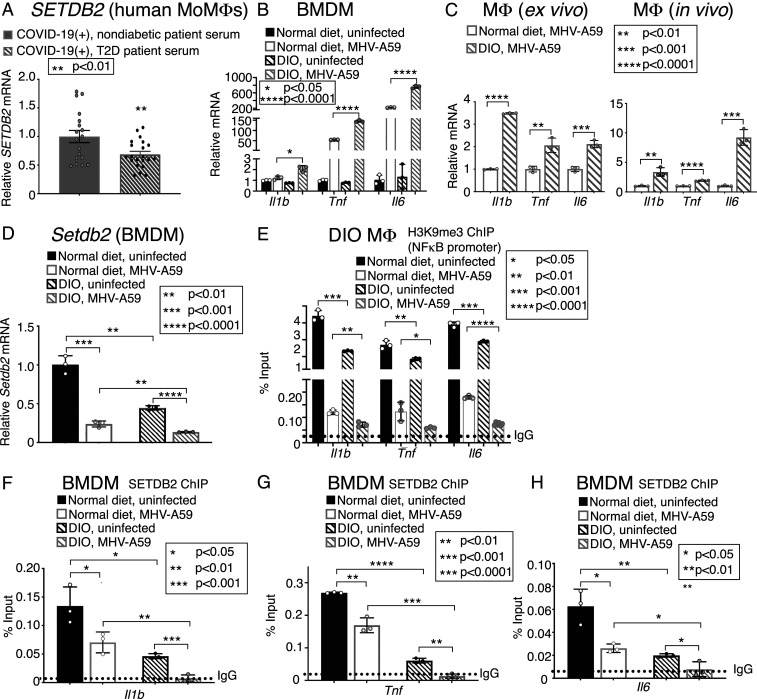
Reduced expression of SETDB2 mediates an increased inflammatory cytokine response in human and murine diabetic Mφs in response to infection with coronavirus SARS-CoV-2 and MHV-A59. (*A*) *SETDB2* expression measured in MoMs from healthy donors (*n* = 3) 24 h following exposure to serum (1:1 diluted in RPMI) from critically ill COVID-19 (+) diabetic (*n* = 21) and nondiabetic (*n* = 18) patients, run in triplicate. (*B*) *Il1b, Tnf,* and *IL6* expression measured in BMDMs from *DIO C57BL/6* mice 24 h following in vitro infection with MHV-A59 (MOI 0.5) and compared to *WT C57BL/6* BMDMs (*n* = 10 mice per group, pooled and run in triplicate). (*C*) *Il1b, Tnf,* and *IL6* expression of splenic Mφs (CD3^−^/CD19^−^/NK1.1^−^/Ly6G^−^/CD11b^+^) from *DIO C57BL/6* mice and *WT* controls 24 h following infection ex vivo with MHV-A59 (MOI 0.5) and after 5 d of in vivo infection with MHV-A59 (2 × 10^5^ pfu) (*n* = 5 mice per group, pooled and run in triplicate). (*D*) *Setdb2* expression measured in measured in BMDMs from *WT* and *DIO C57BL/6* mice 5 h after infection with MHV-A59 (MOI 0.5) (*n* = 5 mice per group, pooled and run in triplicate). (*E*) ChIP analysis of H3K9me3 on the *Il1b, Tnf,* and *IL6* promoters in splenic Mφs (CD3^−^/CD19^−^/NK1.1^−^/Ly6G^−^/CD11b^+^) isolated from *DIO C57BL/6* mice 5 d after intranasal infection with MHV-A59 (2 × 10^5^ pfu) compared to uninfected *DIO* Mφs (*n* = 5 mice per group, pooled and run in triplicate). (*F*–*H*) ChIP analysis of SETDB2 on the *Il1b* (*F*), *Tnf* (*G*), and *Il6* (*H*) promoters in BMDMs from *WT* and *DIO C57BL/6* mice 24 h following in vitro infection with MHV-A59 (MOI 0.5) and compared to uninfected controls (*n* = 5 mice per group, pooled and run in triplicate). *P < 0.05, **P < 0.01, ***P < 0.001, ****P < 0.0001. Data are presented as the mean ± SEM (in *A*) and mean ± SD (in *B*–*H*). All data are representative of two to four independent experiments. Data were first analyzed for normal distribution, and, if data passed the normality test, two-tailed Student’s *t* test was used.

Obesity and T2D are major comorbidities associated with severity of COVID-19 infection, with obese and diabetic patients showing independently higher mortality (3, 16). COVID-19 patients with T2D also demonstrate increased likelihood of a cytokine storm compared to nondiabetics, with a recent study showed elevated serum IL-8 and TNFα levels in these patients, although the mechanisms responsible for this are unclear (3, 16). In order to investigate the increased inflammation in coronavirus associated with diabetics, we first treated human MoMs with sera from T2D and nondiabetic patients hospitalized with COVID-19. The MoMs treated with sera from T2D patients demonstrated decreased *SETDB2* expression compared to treatment with serum from nondiabetic patients with COVID-19 (Fig. 5*A* and *SI Appendix*, Fig. S8). We then examined BMDMs and splenic Mφs (CD3^−^/CD19^−^/NK1.1^−^/Ly6G^−^/CD11b^+^) that were isolated from diet-induced obesity (DIO) mice and infected with MHV-A59. The DIO mouse mirrors human physiology of “prediabetes” in its dietary-induced weight gain, development of insulin resistance, and glucose intolerance compared to normal diet (ND) control mice. Following MHV-A59 infection, these DIO Mφs displayed increased inflammatory cytokine (*IL-1β*, *TNFα*, and *IL-6*) expression compared to nondiabetic controls (Fig. 5 *B* and *C*). Mφs isolated from DIO mice infected with respiratory MHV-A59 (2 × 10^5^ pfu) also demonstrated increased inflammatory cytokine expression compared to nonobese, nondiabetic control mice infected with respiratory MHV-A59 (Fig. 5*C*). Similar to our previous work with wound Mφs showing a decrease of SETDB2 in T2D (22), we found that *Setdb2* in BMDMs was decreased in DIO BMDMs compared to ND controls both at baseline and after MHV-A59 infection (Fig. 5*D*). We found that in vivo Mφs isolated from DIO mice following MHV-A59 infection demonstrated decreased H3K9me3 at NFkB binding sites on the promoter(s) *IL-1β*, *TNFα*, and *IL-6* compared to infected ND Mφs (Fig. 5*E*). Furthermore, SETDB2-ChIP demonstrated decreased SETDB2 at inflammatory gene promoters in DIO BMDMs compared to controls both before and after infection, whereas, after MHV-A59 infection, SETDB2 was undetectable in DIO BMDMs. Taken together, these results indicate that, in obese, diabetic Mφs, loss of *Setdb2* is profound following coronavirus infection and leads to increased inflammatory cytokine production, suggesting a possible mechanism for human T2D patients’ inflammatory response following SARS-CoV-2 infection.

### IFNβ Can Decrease Diabetic Mφ-Mediated Inflammation in Response to Infection with Coronavirus MHV-A59 In Vitro via Up-regulation of SETDB2.

**Fig. 6.**
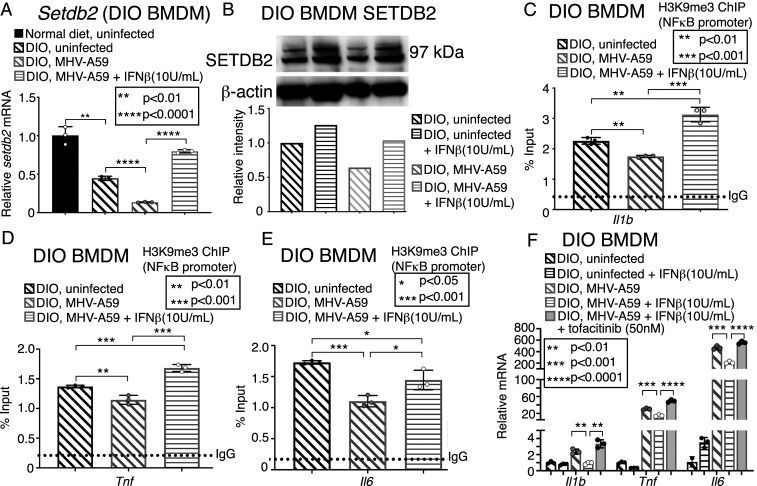
IFNβ decreases diabetic Mφ-mediated inflammation in response to infection with coronavirus MHV-A59 via up-regulation of SETDB2. (*A*) *Setdb2* expression measured in BMDMs from *DIO C57BL/6* mice 5 h following in vitro infection with MHV-A59 (MOI 0.5), with and without coadministration with IFNβ (10 U/mL) (*n* = 5 mice per group, pooled and run in triplicate). (*B*) SETDB2 protein measured in BMDMs from *C57BL/6* mice 12 h following in vitro infection with MHV-A59 (MOI 0.5), with and without coadministration with IFNβ (10 U/mL), via Western blot (*n* = 5 mice per group, pooled). Representative blot is shown. (*C*–*E*) ChIP analysis of H3K9me3 on the *Il1b* (*C*), *Tnf* (*D*), and *Il6* (*E*) promoters in BMDMs from *DIO C57BL/6* mice 24 h following in vitro infection with MHV-A59 (MOI 0.5) with and without coadministration of IFNβ (10 U/mL) compared to uninfected DIO BMDMs (*n* = 5 mice per group, pooled and run in triplicate). (*F*) *Il1b, Tnf,* and *IL6* expression in BMDMs from *DIO C57BL/6* mice 24 h following in vitro infection with MHV-A59 (MOI 0.5), with and without coadministration with tofacitinib (50 nM) and IFNβ (10 U/mL) with tofacitinib (*n* = 5 mice per group, pooled and run in triplicate). *P < 0.05, **P < 0.01, ***P < 0.001, ****P < 0.0001. Data are presented as the mean ± SD. All data are representative of two to four independent experiments. Data were first analyzed for normal distribution, and, if data passed the normality test, two-tailed Student’s *t* test was used.

**Fig. 7.**
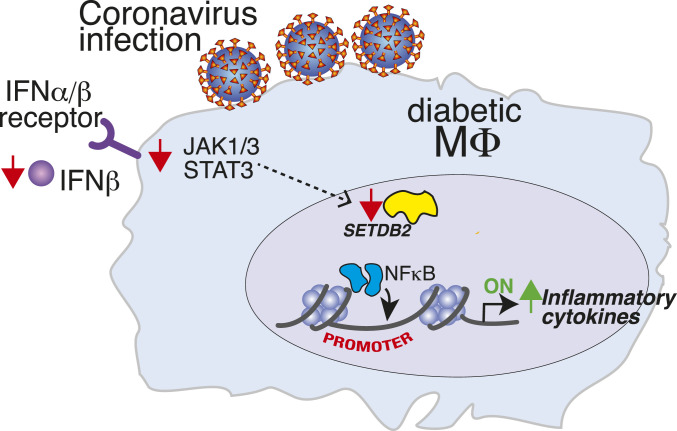
Schematic of SETDB2 in diabetic macrophages following coronavirus infection.

Given that a diminished type I interferon response is implicated in COVID-19 (6, 10), and several clinical trials are underway to determine whether treatment with exogenous IFNβ can improve coronavirus outcomes (37–39), we sought to investigate whether administration of IFNβ could improve Mφ-mediated inflammation, specifically in our obese, diabetic mice following coronavirus infection. Additionally, we identified that levels of IFNβ in the plasma of COVID-19 (+) patients with T2D were significantly decreased compared to non-T2D COVID-19 (+) patients, and that coronavirus-infected T2D human and diabetic (DIO) murine Mφs expressed less *IFNB1* than their non-T2D infected controls (*SI Appendix*, Fig. S9). Thus, BMDMs from DIO mice were administered IFNβ (10 U/mL) during MHV-A59 infection. This led to a significant up-regulation of SETDB2 mRNA and protein compared to untreated but infected BMDMs from DIO mice (Fig. 6 *A* and *B*), and increased *Setdb2* to a significantly greater degree in infected DIO BMDMs compared to infected nondiabetic BMDMs (*SI Appendix*, Fig. S10). Further, we performed a ChIP assay for H3K9me3 on NFkB binding sites on inflammatory gene promoters in DIO BMDMs. Repressive H3K9me3 at the NFkB binding sites of promoter(s) of *Il1b*, *Tnf*, and *Il6* were increased in infected DIO BMDMs coadministered IFNβ (Fig. 6 *C*–*E*). This correlated with a decrease in inflammatory cytokine expression (*Il1b*, *Tnf*, and *Il6*) by DIO Mφs given IFNβ during MHV-A59 infection compared to infected DIO BMDMs not treated with IFNβ (Fig. 6*F*). Furthermore, this antiinflammatory effect was completely nullified in the presence of the JAK1,3 inhibitor tofacitinib, used to block the IFNβ-Setdb2 signaling pathway (Fig. 6*F*). Taken together, these results demonstrate that, in diabetic Mφs, inflammation during coronavirus infection is mediated by SETDB2 via repressive H3K9me3 of NFkB-dependent promoters, and this is, in turn, regulated by IFNβ via a JAK/STAT3 pathway (Fig. 7). Thus, IFNβ administration appears to be an important therapeutic strategy in the diabetic setting for reducing inflammation, in part due to an up-regulation of SETDB2 and repressive H3K9me3 at promoter sites of key inflammatory genes involved in the cytokine storm.

## Discussion

In this study, we identified that SETDB2 is crucial for regulating Mφ-mediated inflammation during coronavirus infection. Down-regulation of SETDB2 following infection led to the loss of H3K9 trimethylation at NFkB binding sites on the promoters of inflammatory genes, leading to increased transcription. Specifically, *Setdb2* expression during coronavirus infection was found to be dependent on IFNβ via JAK1/STAT3 signaling, and disruptions to this pathway led to altered transcription of inflammatory genes. Levels of IFNβ were found to be reduced in plasma from both human COVID-19 (+) T2D patients and MHV-infected diabetic mice as compared to their respective infected nondiabetic patients/mice. Furthermore, treatment of coronavirus-infected Mφs with low doses of IFNβ reduced inflammation via up-regulation of *Setdb2*, particularly in the setting of T2D. Thus, the manipulation of this pathway in Mφs, and possibly in the setting of diabetes, may offer promise as a translational therapy to limit pathologic inflammation and development of the cytokine storm associated with COVID-19.

It has been previously established that SETDB2 is an important regulator of inflammation in Mφs during wound repair and that SETDB2 is decreased at baseline in diabetic wounds (22). Although the role of SETDB2 in Mφs in response to coronavirus is unknown, during influenza infection, up-regulation of SETDB2 dampens inflammation leading to enhanced susceptibility to bacterial superinfections (31). A robust T1IFN response during influenza infection also leads to high levels of SETDB2 and repression of important antiviral mediators (18). Here, we identified that, in contrast to influenza, coronavirus induces a decrease in *Setdb2*, leading to unrestricted transcription of inflammatory cytokines and thus, pathologic inflammation. This is important, as this response to coronavirus infection was exaggerated in diabetic Mφs, secondary to decreased SETDB2 at baseline in diabetic Mφs (22) and an early inadequate T1IFN response (6, 10, 36). Coronavirus infection and T2D together represent a “double hit” to decrease SETDB2 expression and function in diabetic Mφs, preventing H3K9me3 at NFkB binding sites of inflammatory gene promoters and allowing for dysregulated inflammation. One limitation of this study is that there are multiple binding sites for NFkB on inflammatory gene promoters, and, although our H3K9me3 and SETDB2-ChIP analyses correlate with cytokine expression at the most significant proximal binding sites (22), it is possible that other H3K9-targeting methyltransferases play a role in regulating inflammatory gene expression in the setting of coronavirus. Additionally, levels of IFNβ were only measured at a single time point during infection, and these levels may fluctuate over the course of infection. Regardless, the data presented herein do demonstrate that regulation of NFkB-mediated transcription by SETDB2 following viral infection is likely critical to achieve a homeostatic response to initial or subsequent pathogenic stimuli.

Although there is robust literature regarding T1IFNs in response to viral infection and in autoimmune disease, very little is known about their role in Mφ-mediated inflammation during coronavirus infection. Clinical trials investigating the therapeutic potential of IFNβ in COVID-19 are ongoing, with promising early results (37–39); and an early inadequate T1IFN response to SARS-CoV-1 is associated with severe infection (36), but the specific mechanisms regarding the association between T1IFN and severe COVID-19 infection are unknown (6, 10, 36). Additionally, patients with in-born defects in T1IFN activity are more susceptible to severe cases of COVID-19 (40). During the SARS-CoV-1 epidemic, studies suggested that dysregulated T1IFN responses culminated in a failure to switch from a hyperinnate immune response to a more protective adaptive response (36, 41). Several studies demonstrated the efficacy of IFNβ in treating SARS-CoV-1 and MERS-CoV in vitro (36, 42, 43) but with mixed results in treating patients with severe coronavirus infections (37, 38, 44, 45), possibly due to nonspecific targeting of cell subtypes, varied timing of administration, or based on specific patient factors (i.e., T2D). Other studies in patients with COVID-19 have shown overactivity of T1IFNs at later time points during a prolonged critically ill state (6). The effects of T1IFNs are pleiotropic in the setting of viral defense and inflammation (34, 35), and clearly dependent on kinetics, as they play different roles in early and late infection (6, 34–36, 44). Patients infected with MERS-CoV who were treated with IFNβ within 7 d of symptom onset had a drastic reduction in mortality, but patients who started treatment later, after 7 d of symptoms, had no significant benefit (45). Thus, the potential therapeutic window of exogenous IFNβ administration in patients with COVID-19 is likely dependent on kinetics and cell- and patient-specific factors.

Previous work examining SETDB2 in influenza (18) and wound repair (22) has found that SETDB2 is dependent on JAK/STAT signaling. We found that this pathway is also active following coronavirus infection, and, specifically, that a JaK1,3 inhibitor, tofacitinib, decreases *Setdb2* expression during coronavirus infection, and blocked the ability of IFNβ to up-regulate *Setdb2*, particularly in diabetic Mφs. Inflammatory cytokine expression in Mφs was also enhanced with JaK inhibition. Furthermore, Mφs deficient in STAT3 displayed decreased *Setdb2* expression and increased inflammatory cytokine expression in response to coronavirus infection. Although studies have shown promise in improving clinical outcomes in COVID-19 with JaK inhibition (46, 47), these studies have not focused on early administration and were not cell-specific therapies. Thus, the current clinical literature remains mixed regarding the impact of JAK signaling on COVID-19. The reason for these mixed results may be due to differences in the timing of administration, lack of cell specificity, coadministration with other immunomodulatory medications like steroids, or the differential effects in specific patient populations (i.e., T2D). For example, the median enrollment of patients in a study investigating JaK inhibition in COVID-19 was 8 d after symptom onset (47), dissimilar to our study where in vivo Mφs were harvested at multiple early time points 3 d to 7 d after infection. Additionally, the T1IFN response in COVID-19 is suggested to have an inadequate early increase (10), but there is also evidence of an overactive, dysregulated response at late times in the lung (48) following infection leading to increased inflammation. Our results seem to be most clinically relevant in early infection, and studies investigating clinical use of IFNβ early in infection and using cell-specific targeting are deserved.

In conclusion, we identified that SETDB2 is a regulator of Mφ-mediated inflammation during coronavirus infection, and that decreases in *Setdb2* following infection lead to unrestricted transcription of inflammatory cytokines. Through IFNβ signaling via a JaK1/STAT3 pathway, SETDB2 regulated NFkB binding sites of inflammatory promoters in Mφs. Our work offers a description of how the cytokine storm develops in coronavirus infection, and suggests therapeutic potential for treatment of COVID-19 in patients with decreased SETDB2 and, potentially, patients with diabetes.

## Materials and Methods

### Mice.

All mice were maintained at the University of Michigan in the Unit for Laboratory and Animal Medicine (ULAM). Mouse experiments were conducted with approval from our institutional animal care and use committee (IACUC), and all regulatory and safety standards were strictly adhered to. C57BL/6 mice were obtained at 6 wk to 7 wk of age from Jackson Laboratory and maintained in breeding pairs at the ULAM facilities. Mice with the SETDB2 gene deleted in myeloid cells (*Setdb2*^*f/f*^*Lyz2*^*Cre+*^) were generated by mating *Setdb2*^*f/f*^ mice with *Lyz2*^*Cre*^ (Jackson Laboratory) mice as previously described (18). Animals were housed in a barrier facility on a 14-h-light/10-h-dark cycle (ambient temperature of 22 °C) with free access to water, food (Lab Supply Lab Diet Rodent 5001), and bedding (Andersons Lab Bedding Bed-o’Cobs combo). Mice infected with respiratory viruses were maintained in a University of Michigan ULAM ABSL-2 facility for the duration of the experiments.

To induce a prediabetic state, male *C57BL/6* mice were maintained on a high-fat diet (60% kcal saturated fat, 20% protein, 20% carbohydrate, Research Diets, Inc.) for 12 wk to 18 wk to induce the DIO model of T2D as previously described (20, 49). After the appropriate period, high-fat diet−fed (DIO) mice developed obesity and insulin resistance with fasting blood sugars in the mid-200s and elevated insulin levels (20, 49). Following IACUC approval, mice underwent experiments at 20 wk to 32 wk of age. Only male mice were used for these experiments, because female mice do not develop DIO on a high-fat diet. The number of mice used per experiment can be found in the figure legend of each corresponding experiment.

### Murine Coronavirus.

MHV-A59 was obtained from K. R. Wigginton, University of Michigan, Ann Arbor, MI, and cultured as previously described (50). Briefly, murine delayed brain tumor (DBT) cells were grown from frozen stocks in Dulbecco’s modified Eagle’s medium (DMEM) (BW12614F, Lonza) with 10% horse serum (26050088, Invitrogen), glutamine, and penicillin/streptomycin, and cultured for multiple passages until 75% confluence was achieved. Following subculture to T175 flasks, MHV-A59 was propagated in DBT cells via an initial infection MOI of 0.01 for 24 h in DMEM media with 2% horse serum. The subsequent viral supernatant was filtered through a 0.22-μm filter and stored at −80 °C, until thawed once for use. Viral counts were performed using an L2 cell plaque assay. Briefly, L2 cells were cultured for multiple passages using the same protocol as murine DBT cells. Following infection for one hour with 200 μL of viral dilution per 12-well plate, inoculum was removed. One milliliter of 50:50 1.6% agarose (BP160-100, Fisher)/2×MEM (507517464, Thermo Fisher) media with 5% horse serum was added to each 12-well plate and incubated for 48 h. Wells were stained with neutral red (N2889, Sigma) for 1 h, and plaques were counted.

### Magnetic Activated Cell Sorting of Murine Macrophages.

Briefly, following splenic morcellation, splenic cell isolates were incubated with fluorescein isothiocyanate (FITC)-labeled anti-mouse anti-CD3 (Research Resource Identifier [RRID]: AB_312660), anti-NK1.1 (RRID: AB_448547), anti-CD19 (RRID: AB_2629813), and anti-Ly6G (BioLegend, RRID: AB470400) monoclonal antibodies conjugated to FITC. Wound isolates were then washed and incubated with anti-FITC microbeads (Miltenyi Biotec, RRID: AB_244371, catalog no. 130-049-601) and passed through a magnetic activated cell sorting (MACS) column (Miltenyi Biotec). The resultant eluent was then incubated with anti-mouse anti-CD11b microbeads, (Miltenyi Biotec, catalog no. 130-049-601). The remaining cell population was analyzed by flow cytometry and found to be 95% macrophages consistent with previous literature (20, 51).

### BMDM Culture.

Femurs and tibias of mice were flushed with Roswel Park Memorial Institute (RPMI) (Lonza) media, and BMDMs were cultured as described previously (20). After initial cell counting and plating in RPMI, 20% fetal bovine serum (FBS), 30% L-cell supernatant, glutamine, and penicillin/streptomycin, cells were cultured for 7 d. Cells were counted and replated in triplicate on day 6 and infected on day 7. When indicated, BMDMs were stimulated on day 7 with lipopolysaccharide (LPS) (10 ng/mL).

### In Vitro, Ex Vivo, and In Vivo MHV-A59 Infection.

BMDMs were infected at an MOI of 0.5 on day 7 postharvest, following replating on day 6. Infection media (500 cc per 24 wells) included thawed virus, RPMI without FBS, glutamine, and penicillin/streptomycin. Following 4 h, inoculum was removed, and 500 cc of RPMI media with 10% FBS was added. For ex vivo infections, freshly isolated splenic macrophages via MACS were counted and plated (5 × 10^5^) per 24 wells, and infected with MHV-A59 at an MOI of 0.5 in the FBS-free infection media described. Following 4 h, 10% FBS was added directly to each well. For respiratory infections of mice with MHV-A59, WT and DIO C57BL/6 mice were maintained in a University of Michigan ULAM ABSL-2 facility for duration of experiments. Mice were anesthetized with ketamine and administered 1 × 10^5^ pfu to 8 × 10^5^ pfu of virus intranasally followed by 20 μL of phosphate-buffered saline (PBS). Mice were euthanized at multiple time points postinfection and splenic macrophages were harvested and isolated as described. Following isolation, they were immediately processed for RNA extraction or ChIP assay of H3K9me3.

### COVID-19 Patient Serum/Plasma.

All experiments using human samples were approved by the Institutional Review Board (HUM00182169) at the University of Michigan and were conducted in accordance with the principles in the Declaration of Helsinki. Informed consent was obtained from each patient prior to sample acquisition for research purposes. Briefly, age-matched sera and/or plasma were collected from critically ill patients either infected with SARS-CoV-2 (*n* = 35) or hospitalized in the ICU for other reasons (*n* = 24).

### Human Monocyte Isolation and Exposure to COVID-19 Sera.

For human monocyte isolation, peripheral blood was collected from hospitalized ICU patients with (*n* = 12) and without (*n* = 7) COVID-19 and also collected from nondiabetic donors (*n* = 4), and buffy coat was isolated via centrifugation. Cell suspensions were then treated with anti-human CD14 microbeads (EasySep Human CD14 Positive Selection Kit, Stemcell Technologies) and purified by MACS as described above. Healthy donor CD14^+^ monocytes were then counted and cultured for 6 d in RPMI, FBS, M-CSF (25μg/mL, R & D systems), glutamine, and penicillin/stretopmycin, generating MoMs. Sera from the COVID-19 patients and their controls were added to 1:1 with fresh media onto the MoMs. RNA was extracted following 24 h of culture.

### Coadministration of Pharmaceutical Agents to In Vitro Infections.

When indicated, BMDMs were stimulated with/without IFNβ (10 U/mL) (PBL Assay Science, catalog no. 12400-01). Likewise, for JAK1,3 inhibition, cells were treated with 50 nM tofacitinib (Cayman Chemicals) at the time of stimulation with IFNβ. Agents were added directly to the infection media at time of infection and removed with the inoculum after 4 h.

### RNA Isolation.

Total RNA extraction was performed with TRIzol (Invitrogen, Thermo Fisher Scientific) using the manufacturer’s directions. RNA was extracted using chloroform, isopropanol, and ethanol. The iScript (Bio-Rad) or SuperScript III Reverse Transcriptase (Thermo Fisher Scientific) kits were used to synthesize complementary DNA (cDNA) from extracted RNA. We used cDNA primers for *Il1b* (Mm00434228_m1), *Tnf* (Mm00443258_m1), *Il6* (Mm00446190_m1), *IFNB1* (Hs01077958_s1, Mm00439552_s1), and *SETDB2* (Mm01318752_m1, Hs01126272), and used 18s or glyceraldehyde-3-phosphate dehydrogenase (GAPDH) as the internal control. Data were analyzed relative to 18S ribosomal RNA or GAPDH (2^−ΔCt^). All samples were assayed in triplicate. The threshold cycle values were used to plot a standard curve. Data are representative of two or three independent experiments and were compiled in Microsoft Excel and presented using Prism software (GraphPad).

### ELISA.

Mouse inflammatory cytokine (IL-1β, TNFα, and IL-6) and human IFNβ concentration was measured by ELISA kits (D4410-05, R&D systems) per the manufacturer’s protocol. Color intensity was measured at 450 nm. The ELISA kit has a detection limit of 15.6 pg/mL.

### Western Blot.

Cell suspensions were lysed in radioimmunoprecipitation assay (RIPA) buffer (SIGMA) and standardized for protein concentrations using a Bradford protein assay (BioRad) to generate a standard curve. Equal amounts of protein were then loaded onto to 4 to 12% sodium dodecyl sulfate gel electrophoresis under reducing conditions. Protein bands were then transferred to polyvinylidene difluoride (PVDF) membranes and probed with primary antibodies (anti-human SETDB2 [RRID: AB_2855768, Invitrogen], anti-mouse beta actin [RRID: AB_2855768, Invitrogen]) at 4 °C for 12 h. All primary antibodies were diluted 1:500 in 5% BSA in 0.1% Tween Tris-buffered saline (TBS-T) solution. PVDF membranes were then washed and incubated with horseradish peroxidase−labeled secondary antibody (Cell Signaling, Inc.) for 1 h at room temperature and visualized with chemiluminescence (Thermo Fisher Scientific). Blot images were analyzed using NIH ImageJ software to obtain sample densitometry readings normalized to beta actin.

### SETDB2 Immunofluorescence Microscopy.

Murine BMDMs were plated on coverslips and infected, as described, at MOI 0.5. Following 12 h of infection, coverslips were fixed in 2% paraformaldehyde for 30 min at room temperature, washed twice with PBS, and permeabilized with 1% saponin with 0.1% bovine serum albumin (Sigma). Anti-SETDB2 (RRID: AB_ 2850355, Invitrogen) was diluted to 1:500 in the permeabilization buffer and left to stain overnight. Slides were washed twice with PBS and then stained with anti-rabbit Alexa-Flour 568 (RRID: AB_143157, Invitrogen), washed twice more with PBS, and imaged at 20× with a fluorescent microscope (Olympus) exciting Texas Red.

### ChIP Assay.

ChIP assay was performed as described previously (20, 52). Briefly, cells were fixed in 1% paraformaldehyde and lysed and sonicated using a Bioruptor Pico (Diagenode) to generate 300- to 500-bp fragments. Samples were then incubated overnight in anti-H3K9me3 antibody (RRID: AB_306848, ab8898, Abcam), anti-SETDB2 (RRID: AB_ 2850355, Invitrogen), or isotype control (rabbit polyclonal IgG ab171870, Abcam) in parallel followed by addition of protein A-Sepharose beads (Thermo Fisher Scientific). Beads were washed and bound; DNA was eluted and purified using phenol/chloroform/isoamyl alcohol extraction followed by ethanol precipitation. H3K9me3/SETDB2/IgG deposition was measured by qPCR using 2× SYBR PCR mix (Invitrogen, Thermo Fisher Scientific) and primers targeting NFκB binding sites in the *Il1b*, *Tnf*, and *IL6* promoters. Primers were designed using the Ensembl genome browser to search the IL1β, TNFα, and IL6 promoters for NFκB within the promoter region, and then National Center for Biotechnology Information Primer-BLAST was used to design primers that flank this site. The following primers were used to amplify DNA in samples:

IL1β: 5′-GCA​GGA​GTG​GGT​GGG​TGA​GT-3′ and 5′-CAG​TCT​GAT​AAT​GCC​AGG​GTG​C-3′.
TNFα: 5′-TCC​TGA​TTG​GCC​CCA​GAT​TG-3′ and 5′-TAG​TGG​CCC​TAC​ACC​TCT​GT-3′.
IL6: 5′-AGG​TTT​CCA​ATC​AGC​CCC​AC-3′ and 5′-GGG​CTC​CAG​AGC​AGA​ATG​AG-3′.

### Statistics.

GraphPad Prism software (RRID: SCR_002798) version 7.0 was used to analyze the data. Data were analyzed for normal distribution, and then statistical significance between multiple groups was determined using a one-way ANOVA test followed by Newman−Keuls post hoc test. For all single group comparisons, if data passed the normality test, we used a two-tailed Student’s *t* test. Otherwise, data were analyzed using the Mann–Whitney *U* test. All data are representative of at least two independent experiments as detailed in the figure legends. A *P* value of less than or equal to 0.05 was significant.

## Supplementary Material

Supplementary File

## Data Availability

All study data are included in the article and *SI Appendix*.
