# Supplementary material for: Coronavirus induces diabetic macrophage-mediated inflammation via SETDB2
Source: Proc Natl Acad Sci U S A. 2021 Sep 3;118(38):e2101071118. doi: 10.1073/pnas.2101071118 (PMC8463849; doi:10.1073/pnas.2101071118)
Supplement: Supplementary File [file pnas.2101071118.sapp.pdf]

Title Page

Coronavirus Induces Diabetic Macrophage-mediated Inflammation via SETDB2

Authors

William J. Melvin, MD[1]; Christopher Audu, MD, PhD[1]; Frank M. Davis, MD[1]; Sriganesh Sharma, MD, PhD[1]; Amrita Joshi PhD[1]; Aaron DenDekker, PhD[1]; Sonya Wolf, PhD[1]; Emily Barrett, MD[1]; Kevin Mangum, MD,PhD[1]; Xiaofeng Zhou, PhD[2]; Monica Bame, PhD[2]; Alex Ruan[1], Andrea Obi, MD[1]; Steven Kunkel, PhD[3]; Bethany B. Moore, PhD[2,4]; Katherine A. Gallagher, MD*[1,4]

Affiliations

[1]Section of Vascular Surgery, Department of Surgery, University of Michigan, Ann Arbor, MI 48109.
[2]Department of Internal Medicine, University of Michigan, Ann Arbor, MI 48109.
[3]Department of Pathology, University of Michigan, Ann Arbor, MI 48109
[4]Department Microbiology and Immunology, University of Michigan, Ann Arbor, MI 48109.

***Corresponding Author:**
Dr. Katherine A. Gallagher
University of Michigan
Department of Surgery Section of Vascular Surgery
Department of Microbiology and Immunology
5364 Cardiovascular Center
1500 E. Medical Center Drive
Ann Arbor, MI 48109-5867
Telephone: (734) 936-5820
Email: kgallag@med.umich.edu

Author Contributions: K.A.G. and W.J.M. designed the experiments. W.J.M., C.A., A.J., F.M.D, A.D., S.W., S.S., A.O., X.Z., E.B., K.M., M.B., B.B.M., and K.A.G. performed experiments. W.J.M., C.A., A.J., F.M.D, A.D., S.W., S.S., E.B., K.M., A.O., X.Z., M.B., B.B.M., and K.A.G. analyzed data. W.J.M., C.A., A.J., F.M.D, A.D., S.W., S.S., A.O., X.Z., M.B., S.K., B.B.M., and K.A.G. prepared the manuscript.

Competing Interest Statement: The authors declare no competing interest.

Classification: Biological Sciences, Immunology and Inflammation

Keywords: Coronavirus, Diabetes, Inflammation, Epigenetics, Monocyte/Macrophage.

This PDF file includes: Supplemental Figures S1 to S10

Supplemental Figure 1

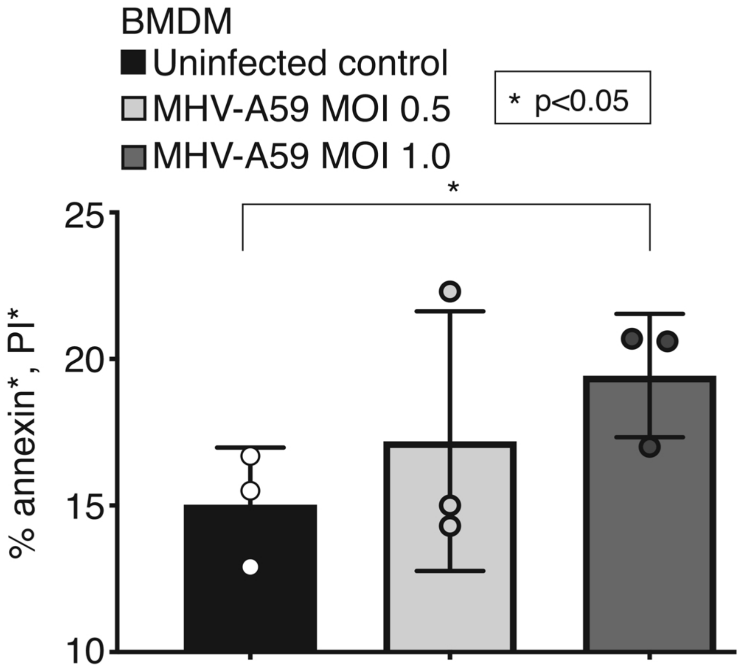

Fig S1. The murine coronavirus MHV-A59 causes increased BMDM cellular death at MOI 1.0. BMDMs from *C57BL/6* mice were infected with MHV-A59 at MOI 0.5 and MOI 1.0 and cell suspensions were analyzed for Annexin-FITC and Propidium Iodide staining by flow cytometry after 5 hours of infection (n=5 mice group, pooled and ran in triplicate).

Supplemental Figure 2

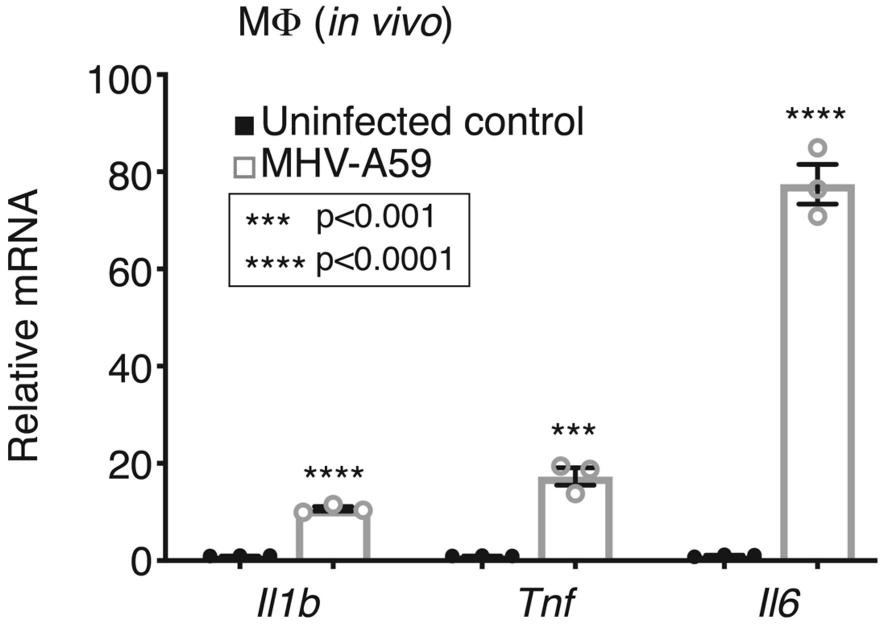

Fig. S2. The murine coronavirus MHV-A59 induces Mφ-mediated inflammation. *Il1b, Tnf*, and *Il6* expression measured in splenic Mφs ($CD3^-/CD19^-/NK1.1^-/Ly6G^-/CD11b^+$) isolated from *C57BL/6* mice 7 days after intranasal infection with MHV-A59 ($2x10^5$ PFU) compared to uninfected Mφs (n=5 mice / group, pooled and run in triplicate).

Supplemental
Figure 3

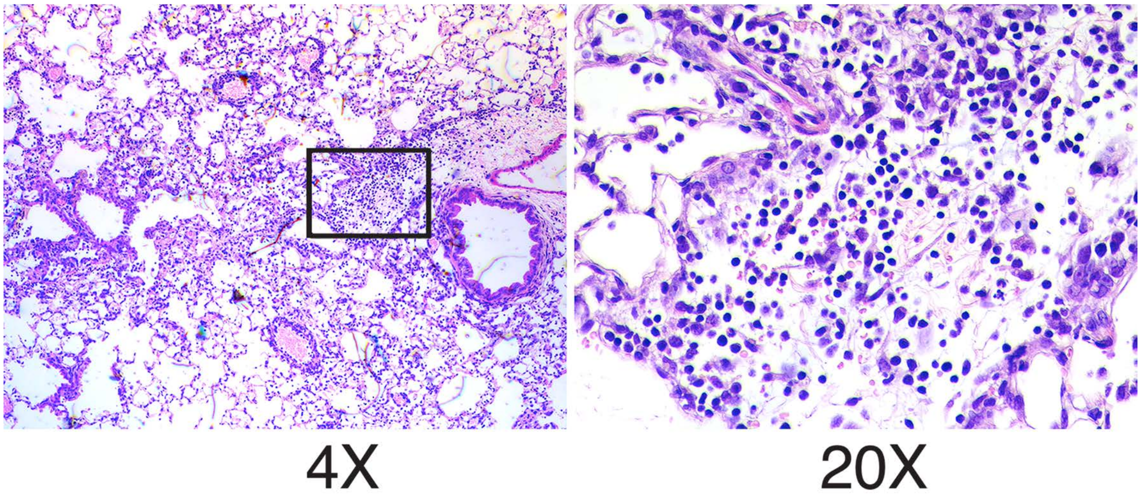

Fig. S3. Representative lung histology in *C57BL/6* mice on day 5 post-infection with the murine coronavirus MHV-A59 (2x10^5 PFU). Pulmonary vasculature was flushed with PBS upon harvest and then whole lungs were fixed with 10% formalin, sectioned, and stained with H&E.

Supplemental
Figure 4

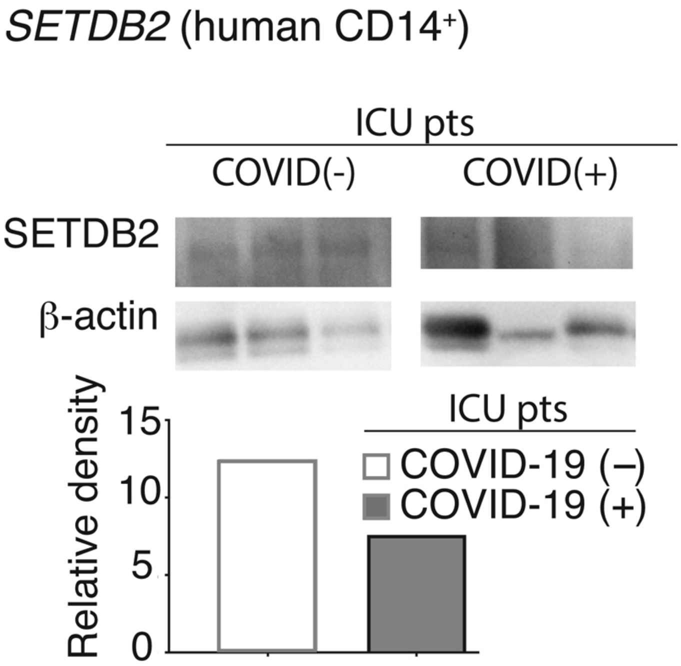

Fig S4. SETDB2 protein measured in human CD14$^+$ cells sorted from peripheral blood of critically ill patients with and without COVID-19 (n=6) by western blot. Representative blot is shown.

Supplemental
Figure 5

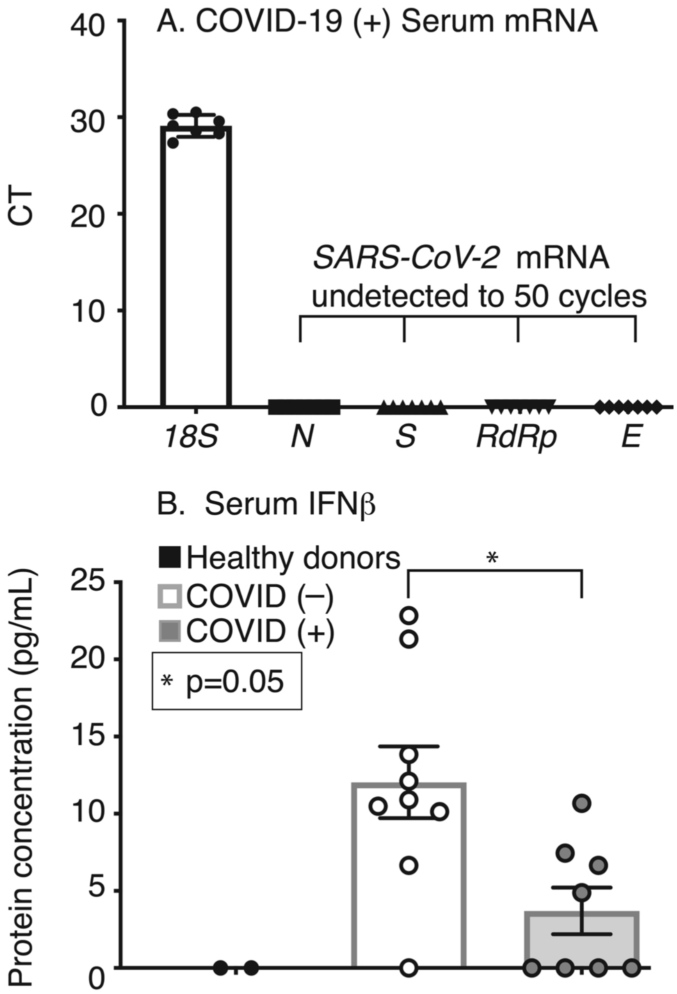

Fig S5. Sera from COVID-19 (+) patients do not have detectable *SARS-CoV-2* mRNA and have decreased IFNβ. (5A) *18S* and *SARS-CoV-2* mRNA (*N, S, RdRp*, and *E*) from COVID-19 (+) sera was measured (n=7). (5B) IFNβ protein in sera from COVID-19 (+) (n=8), COVID-19 (-) (n=9) patients and from healthy donors (n=2) was measured.

Supplemental
Figure 6

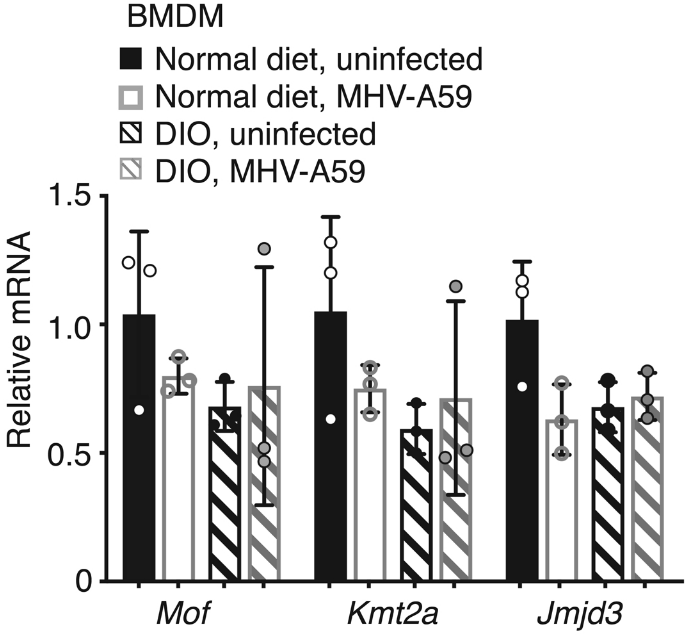

Fig S6. Expression of chromatin modifying enzymes in response to MHV-A59 in normal and diabetic BMDMs. BMDMs from *WT* and *DIO C57BL/6* mice were infected with MHV-A59 at MOI 0.5 and analyzed for expression of *Mof, Kmt2a (Mll1)*, and *Jmjd3* 5 hours after infection (n=5 mice / group, pooled and run in triplicate).

Supplemental
Figure 7

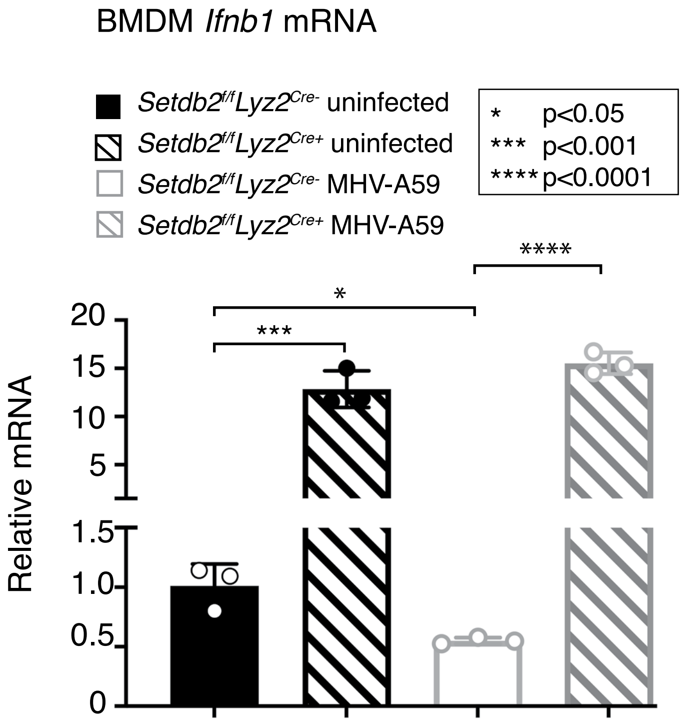

Fig S7. Expression of Ifnb1 is increased in Setdb2-deficient BMDMs. BMDMs from *Setdb2$^{f/f}$Lyz2^{Cre+}* and *Setdb2$^{f/f}$Lyz2^{Cre-}* littermates were infected with MHV-A59 (MOI 0.5), and *Ifnb1* expression was analyzed after 5 hours (n=5 mice / group, pooled and run in triplicate).

Supplemental
Figure 8

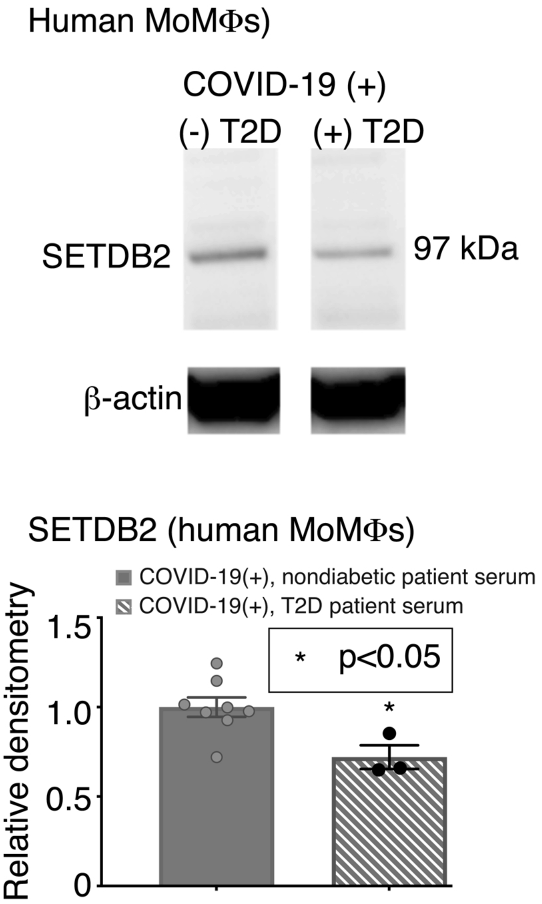

Fig S8. SETDB2 protein is further decreased in human MoMs treated with serum from diabetic COVID-19(+) patients. SETDB2 protein from cell lysates from MoMs from healthy donors (n=3) 24 hours following exposure to serum (1:1 diluted in RPMI) from diabetic COVID-19 (+) (n=3) and nondiabetic COVID-19 (+) (n=8) critically ill patients were measured by western blot. Representative blot shown.

Supplemental
Figure 9

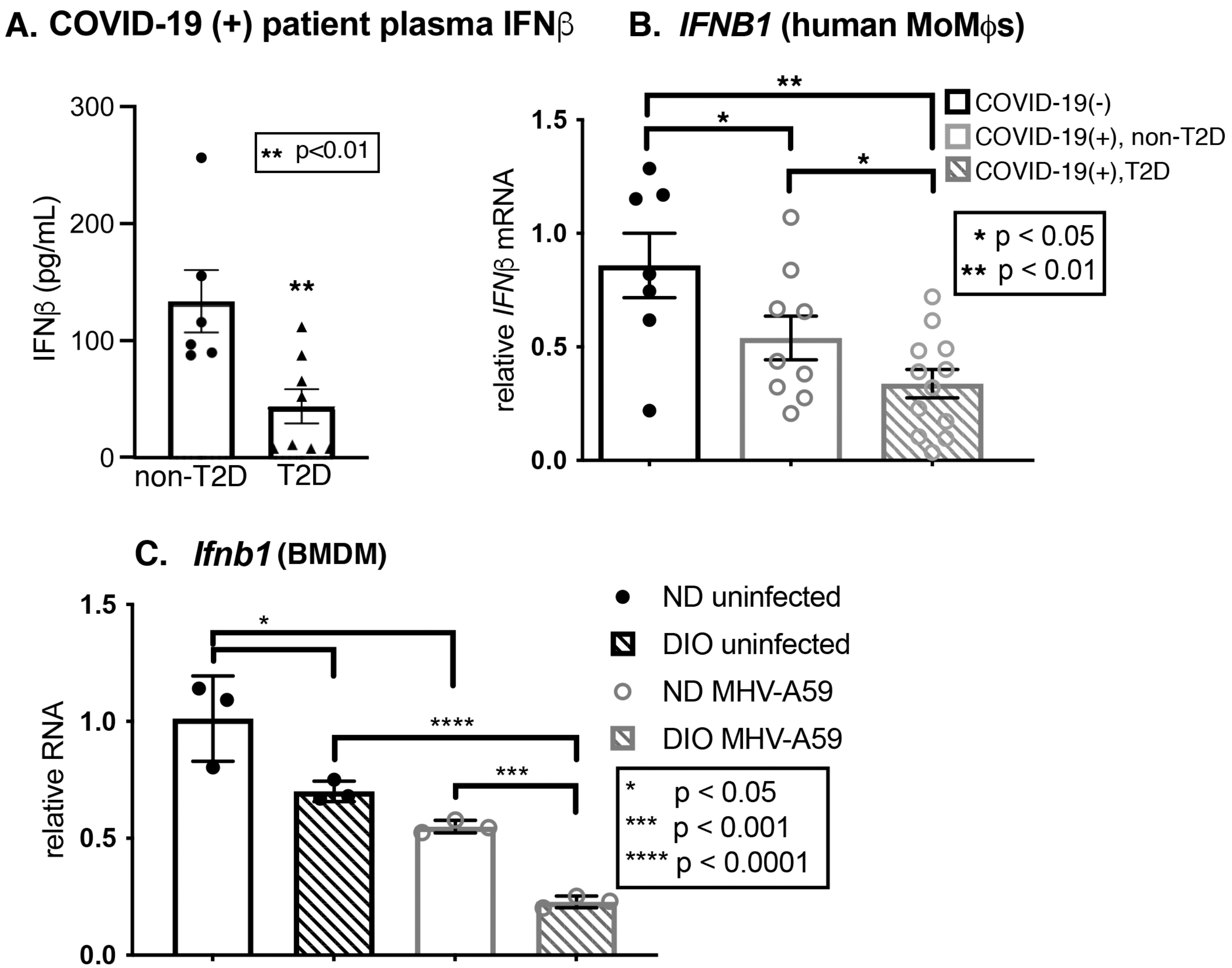

Fig S9. Plasma IFNβ is decreased in T2D patients with COVID-19 compared to non-T2D infected patients, and *IFNB1* expression is decreased in infected T2D human and murine Mφs. (9A) Undiluted plasma from hospitalized patients with COVID-19 with T2D (n=8) and non-T2D (n=6) was measured for IFNβ by ELISA. (9B) *IFNB1* expression measured in MoMs from healthy donors (n=3) 24 hours following exposure to serum (1:1 diluted in RPMI) from COVID-19 (-) (n=7), non-T2D COVID-19 (+) (n=9), and T2D COVID-19 (+) (n=12) critically ill patients. (9C) *Ifnb1* expression measured in BMDMs from WT and DIO *C57Bl/6* mice 5 hours following *in vitro* infection with MHV-A59 (MOI 0.5) (n=5 mice / group, pooled and run in triplicate).

Supplemental
Figure 10

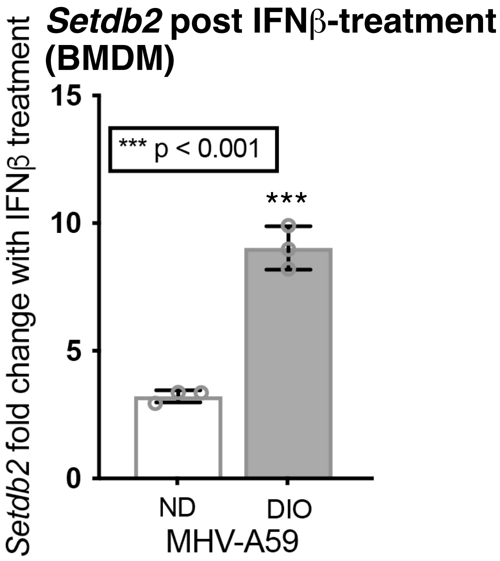

Fig S10. IFNβ treatment of infected murine DIO BMDMs increases *Setdb2* expression to a greater degree compared to treated, infected WT controls. BMDMs from *WT* and *DIO C57BL/6* mice were infected with MHV-A59 (MOI 0.5) and treated with IFNβ (10U/mL), and *Setdb2* expression was analyzed after 5 hours (n=5 mice / group, pooled and run in triplicate).
